# Furthering Cannabis Research and Education: An Interview with Fundación CANNA

**DOI:** 10.1089/can.2016.29006.svb

**Published:** 2016-05-01

**Authors:** Jordan L. Schilling, Susan van Brunschot

**Affiliations:** ^1^ Mary Ann Liebert, Inc., New Rochelle, New York.; ^2^ Fundación CANNA, Barcelona, Spain.

**Keywords:** cannabinoids, cannabis education, cannabis testing, medicinal cannabis

## Abstract

Fundación CANNA is a nonprofit initiative of CANNA, a multinational company producing high-quality fertilizers for fast growing plants. The Foundation carries out studies and conducts research on cannabis and its active compounds. Fundación CANNA supports different congresses, initiatives, and scientific studies to push forward the knowledge about cannabinoids as a medicine.

***Cannabis and Cannabinoid Research*** **(Jordan L. Schilling:** ***CCR*): To start off, could you tell our readers about the history and mission of Fundación CANNA?**

**Susan van Brunschot (SVB):** Fundación CANNA is a nonprofit organization founded 5 years ago by CANNA, a multinational company producing high-quality fertilizers for fast growing plants. Our headquarters are located in Barcelona, Spain. Also, Fundación CANNA owns a laboratory where we can perform cutting-edge research on cannabis and its compounds, and run different kinds of analytical chemistry tests.

The idea behind Fundación CANNA was conceived while considering the need to answer a broad demand for quality control cannabis testing and expanding the research to address scientific issues. Specifically, we focus on furthering studies and scientific research regarding cannabis's effects on the human body and mind.

The ongoing mission of Fundación CANNA is to inform the public and to support the medicinal cannabis movement worldwide. We plan to continue publishing new articles in collaboration with scientific researchers and distinguished members of the cannabis community.

***CCR*: What kind of educational materials do you provide on your website?**

**SVB:** It is important for us to share knowledge about the cannabis plant with a focus on the endocannabinoid system in humans and all related topics. We spend a lot of energy into making medicinal cannabis users aware about the importance of testing cannabis, oils, and derivatives with scientific methods. There is still a lot of education needed for the public on how cannabis and the endocannabinoid system work in the human body, and the potential therapeutic use of cannabinoids in medicine.

Our website provides various articles about cannabinoids, terpenes, flavonoids, the endocannabinoid system, reports regarding the dangers of contaminants such as heavy metals, but also nutritional benefits of hemp seeds, and so on. Also, readers can find studies that we helped finance and organize regarding health and quality of life of medicinal cannabis users, microbiological contamination studies, methods to quantify cannabinoids, and variations in terpene profiles in different strains of cannabis. Also, we have been looking into the differences in cannabinoid and terpenoid concentration of different cannabis derivatives.

We recently started a Facebook and Twitter account for Fundación CANNA to reach out to the general public, and medicinal cannabis users, to provide them with quality information and address the abundance of misleading information that is available on social media.

***CCR*: What kind of cannabis testing does Fundación CANNA provide?**

**SVB:** In our laboratory, we carry out many different tests such as cannabinoid profiling to determine potency, terpene profiling, and check for contaminants such as solvent residues, heavy metals, and microbiological contamination.

Specifically, we use high-performance liquid chromatography (HPLC) for cannabinoids. Liquid chromatography does not use heat, so it is possible to identify and quantify both acidic and neutral cannabinoids. We believe this is the most accurate method because it shows the real content of cannabinoids present in a sample of cannabis. The neutral cannabinoids that we can qualify and quantify by using HPLC are cannabidiol (CBD), cannabigerol (CBG), tetrahydrocannabinol (THC), and cannabinol (CBN). We can qualify and quantify the following acidic cannabinoids: CBDA, CBGA, and THCA.

The analysis of terpenes allows us to choose those plants that better suit our needs, as well as, based on the proportion of terpenes present, being able to identify and characterize varieties in a more accurate way than by simply assigning names or defining them as Indica or Sativa. Fundación CANNA works with methods of analysis previously validated for the analysis of terpenes by gas chromatography (GC-FID), which enables us to provide accurate and reliable information about the amount present for each of them.

Cannabis oils and extracts have become pretty popular worldwide. However, because of a lack of regulation or standards, we often find contaminations, solvent residues, or other impurities inside the oils. Therefore, Fundación CANNA will start to offer specific tests to determine whether solvent residues are present in a sample. We use headspace gas chromatography with flame ionization detector (HS-GC-FID) to quantify harmful solvents down to parts-per-million (PPM).

Mold and bacteria can be found everywhere. Some of these microorganisms are harmful for humans. Cannabis, like any other product, is susceptible to containing microorganisms acquired during cultivation, drying, processing, handling, and conservation. It is possible to know the total amount of microorganisms present in a sample by performing specific microbiological tests, which give us an idea of its quality. It is also possible to identify microorganisms that can cause severe damage to human health such as *Escherichia coli*. Parameters that are measured include total aerobic microorganisms, total fungi, and yeasts, enterobacteria.

***CCR*: Are your cannabis testing services for a research setting or for both research- and patient-oriented settings?**

**SVB:** Our testing services are both for our own internal studies and for companies that wish to test their medicinal cannabis-based products. However, we provide testing services only within Europe. We are convinced that cannabis should be tested to ensure safer user access and to guarantee its quality to consumers, which should be the aim of all companies and entities active in this market. We are able to use test results to promote internal studies about cannabis and identify trends within the market.

***CCR*: What are some of the research collaborations in which the foundation participates?**

**SVB:** Fundación CANNA has been supporting different scientific associations, and events such as the International Association for Cannabinoid Medicines (IACM), International Cannabinoid Research Society (ICRS), Spanish Society on Cannabis Investigation (SEIC), and other partners worldwide. Last year, we coproduced the documentary “The Scientist” that explored the life of Dr. Ralphael Mechoulam. It was awarded several prizes and achieved international recognition.

All funds collected by donations and commercial laboratory testing service are reinvested in supporting initiatives related to medicinal cannabis and funding new research worldwide. If you are interested in the activities of Fundación CANNA, would like to propose a new study, or would like to generously contribute to help us achieve our goals, please visit our website www.fundacion-canna.es or e-mail: info@fundacion-canna.es

